# Supplementary material for: COZOID: contact zone identifier for visual analysis of protein-protein interactions
Source: BMC Bioinformatics. 2018 Apr 6;19:125. doi: 10.1186/s12859-018-2113-6 (PMC5889581; doi:10.1186/s12859-018-2113-6)
Supplement: Supplementary file 2 — Software build. Executable binary file of the software tool. (ZIP 118,784 kb) [file 12859_2018_2113_MOESM2_ESM.zip › cozoid/java/docs/beansbinding-1.2.1-doc.zip!/org/jdesktop/el/class-use/VariableMapper.html]

Uses of Class org.jdesktop.el.VariableMapper (Beans Binding)


---


|  |  |  |
| --- | --- | --- |
| **Overview** \| **Package** \| **Class** \| **Use** \| **Tree** \| **Deprecated** \| **Index** \| **Help** | |  |
| PREV   NEXT | **FRAMES**    **NO FRAMES**     **All Classes** |  |


---


## **Uses of Class org.jdesktop.el.VariableMapper**

| Packages that use VariableMapper | |
| --- | --- |
| **org.jdesktop.el** | Customized version of EL for Beans Binding; not for general use. |
| **org.jdesktop.el.impl** | Customized version of EL for Beans Binding; not for general use. |
| **org.jdesktop.el.impl.lang** | Customized version of EL for Beans Binding; not for general use. |

Uses of VariableMapper in org.jdesktop.el

| Methods in org.jdesktop.el that return VariableMapper | |
| --- | --- |
| `abstract  VariableMapper` | `ELContext.getVariableMapper()`             Retrieves the `VariableMapper` associated with this `ELContext`. |

Uses of VariableMapper in org.jdesktop.el.impl

Constructors in org.jdesktop.el.impl with parameters of type VariableMapper

`MethodExpressionImpl(java.lang.String expr,
Node node,
FunctionMapper fnMapper,
VariableMapper varMapper,
java.lang.Class expectedType,
java.lang.Class[] paramTypes)`

`ValueExpressionImpl(java.lang.String expr,
Node node,
FunctionMapper fnMapper,
VariableMapper varMapper,
java.lang.Class expectedType)`

Uses of VariableMapper in org.jdesktop.el.impl.lang

| Subclasses of VariableMapper in org.jdesktop.el.impl.lang | |
| --- | --- |
| `class` | `VariableMapperFactory` |
| `class` | `VariableMapperImpl` |

| Methods in org.jdesktop.el.impl.lang that return VariableMapper | |
| --- | --- |
| `VariableMapper` | `VariableMapperFactory.create()` |
| `VariableMapper` | `EvaluationContext.getVariableMapper()` |

Constructors in org.jdesktop.el.impl.lang with parameters of type VariableMapper

`EvaluationContext(ELContext elContext,
FunctionMapper fnMapper,
VariableMapper varMapper,
Expression expression)`

`EvaluationContext(ELContext elContext,
FunctionMapper fnMapper,
VariableMapper varMapper,
Expression expression,
boolean trackResolvedProperties)`

`VariableMapperFactory(VariableMapper target)`

---


|  |  |  |
| --- | --- | --- |
| **Overview** \| **Package** \| **Class** \| **Use** \| **Tree** \| **Deprecated** \| **Index** \| **Help** | |  |
| PREV   NEXT | **FRAMES**    **NO FRAMES**     **All Classes** |  |


---
